# Co-expression of truncated and full-length tau induces severe neurotoxicity

**DOI:** 10.1038/mp.2015.228

**Published:** 2016-02-02

**Authors:** S Ozcelik, F Sprenger, Z Skachokova, G Fraser, D Abramowski, F Clavaguera, A Probst, S Frank, M Müller, M Staufenbiel, M Goedert, M Tolnay, D T Winkler

**Affiliations:** 1 Institute of Pathology, University Hospital Basel, Basel, Switzerland; 2 Department of Neurology, University Hospital Basel, Basel, Switzerland; 3 MRC, Laboratory of Molecular Biology, Cambridge, UK; 4 Institute of Biomedical Research, Novartis Pharma AG, Basel, Switzerland

## Abstract

Abundant tau inclusions are a defining hallmark of several human neurodegenerative diseases, including Alzheimer's disease. Protein fragmentation is a widely observed event in neurodegenerative proteinopathies. The relevance of tau fragmentation for the neurodegenerative process in tauopathies has yet remained unclear. Here we found that co-expression of truncated and full-length human tau in mice provoked the formation of soluble high-molecular-weight tau, the failure of axonal transport, clumping of mitochondria, disruption of the Golgi apparatus and missorting of synaptic proteins. This was associated with extensive nerve cell dysfunction and severe paralysis by the age of 3 weeks. When the expression of truncated tau was halted, most mice recovered behaviorally and functionally. In contrast, co-expression of full-length tau isoforms did not result in paralysis. Truncated tau thus induces extensive but reversible neurotoxicity in the presence of full-length tau through the formation of nonfilamentous high-molecular-weight tau aggregates, in the absence of tau filaments. Targeting tau fragmentation may provide a novel approach for the treatment of human tauopathies.

## Introduction

Tau pathology is a defining characteristic of a number of human neurodegenerative diseases, including Alzheimer's disease, progressive supranuclear palsy, corticobasal degeneration, argyrophilic grain disease, chronic traumatic encephalopathy and some cases of frontotemporal dementia.^1, 2^ Physiologically, tau promotes microtubule assembly and stability. In tauopathies, soluble tau assembles into insoluble filaments, resulting in neurodegeneration.^2, 3, 4^ It remains to be determined which tau species are the most toxic and how toxicity is mediated.

Abnormal protein aggregation underlies the vast majority of human neurodegenerative diseases.^5^ In some diseases, the aggregates are made of cleavage products of larger proteins, such as Aβ in Alzheimer's disease^6^ and amyloid-Bri in familial British dementia.^7^ In other diseases, full-length and truncated proteins co-exist in the aggregates, as is the case of α-synuclein in Parkinson's disease and dementia with Lewy bodies^8, 9^ and TDP-43 in cases of frontotemporal dementia and amyotrophic lateral sclerosis.^10, 11^ Truncated proteins are often more aggregation prone than their full-length counterparts.^12, 13^

It is being increasingly debated whether tau fragmentation may play a role in the pathogenesis of Alzheimer's disease. Cleaved tau has been detected in patient brains and in mouse models.^14, 15, 16, 17, 18, 19, 20^ In Alzheimer's disease, tau fragmentation has been described as an early event. Caspases and calpains have been implicated, with the caspase 3 cleavage after D421 being the most studied.^21^ More recently, asparagine endopeptidase has also been shown to cleave tau and promote pathology.^22^ However, the general relevance of tau fragmentation for neurodegeneration has been questioned. Thus, in mouse lines transgenic for human mutant P301S^23^ or P301L^24^ tau, cleavage after D421 was a late event and only small amounts of caspase-cleaved tau were detected. Moreover, earlier studies found tau fragmentation to be primarily associated with degradation of the fuzzy filament coat.^25, 26^ Full-length tau is the major component of the paired helical and straight filaments of Alzheimer's disease.^27^ On the other hand, truncation of tau increases its propensity to aggregate and it has been suggested that cleaved tau may seed the aggregation of the full-length protein.^28, 29, 30^ Taken together, it is therefore possible that truncation of a small amount of tau can lead to its aggregation and the seeding of full-length tau.

Here we studied the interaction of truncated and full-length human tau. We generated an inducible mouse line (TAU62) overexpressing human 3R tau_151–421_ (Δtau). This 239 amino acid tau protein extends from the proline-rich region to the caspase cleavage site. We co-expressed it with either wild-type full-length 3R tau or 4R tau,^31^ or with mutant full-length 4R P301S tau.^32^ In all double transgenic lines, high-molecular-weight tau, severe nerve cell damage and motor palsy were observed in young mice. Following cessation of truncated tau expression, functional and structural recovery was observed in mice expressing full-length 4R tau. In contrast, young mice double transgenic for full-length 3R and 4R tau were unaffected.

## Materials and Methods

### Production of transgenic mouse lines and doxycycline treatment

An overview of the mouse lines used in this study is provided in Supplementary Table 1. For the neuron-specific, inducible expression of 3R tau_151–421_ (Δtau), TAU62 transgenic mice were generated by coinjection of two Thy 1.2 minigene-based^33^ constructs into C57BL/6J oocytes. The Thy1.2-tTS construct was obtained by inserting a tetracycline-controlled transcriptional silencer element (tTS) complementary DNA into the *Xho*I site of the murine Thy 1.2 minigene. The Thy1.2-TRE-Δtau construct contained a tetracycline-responsive element (TRE) in the *Spe*I site of the Thy1.2 cassette ∼860 bp upstream of human wild-type Δtau complementary DNA encoding amino acids 151 to 421 of a 3-repeat domain spanning human wild-type tau fragment (0N3R tau_151–421_) cloned in the *Xho*I site. Six transgenic founder TAU62 mice (C57BL/6J-TgN(TRE-Thy1tau_151–421_xThy1tTS)62) were identified and the inducible expression of human Δtau was assessed by western blotting and immunohistochemistry. Lines 62/2 and 62/48 expressed similar levels of Δtau (‘*on*') and stopped expression following the removal of doxycycline (‘*on-off*'). Most experiments were performed using the TAU62/48 line, abbreviated TAU62. TAU62/2 mice were used to rule out an insertion site effect. The production of P301S mutant 0N4R tau transgenic mice (C57BL/6J-TgN(Thy1-hTau_P301S_))^32^ and full-length wild-type 2N4R tau transgenic ALZ17 mice (C57BL/6J-TgN(Thy1hTau)17) has been previously described.^31^ For the generation of ALZ31 wild-type human 0N3R tau transgenic mice (C57BL/6J-TgN(Thy1hTau)31), 0N3R human tau complementary DNA was cloned into the Thy 1.2 minigene and injected into C57BL/6J oocytes. P301SxTAU62, ALZ17xTAU62 and ALZ31xTAU62 double transgenic mice were obtained by crossbreeding of the respective single transgenic lines using TAU62/48 mice if not indicated otherwise. All transgenic mice, including P301SxALZ31 and ALZ17xALZ31 mice, were heterozygous for the transgenes of interest, unless specifically mentioned otherwise. Food containing 500 mg kg^−1^ doxycycline was provided *ad libitum* also during breeding to induce Δtau expression. The number of mice used was minimized according to the Swiss regulation on Animal Experimentation. All animal experiments were approved by the local ethics and animal care and use committees.

### Histology and immunohistochemistry

Mice were anesthetized with a mixture of ketamine (100 mg kg^−1^) and xylazine (10 mg kg^−1^) intraperitoneally and after deep sleeping, mice were injected by sodium pentobarbital (100 mg kg^−1^) and transcardially perfused with cold phosphate-buffered saline (PBS). Spinal cord, sciatic nerve and the brain were quickly removed. Brain and spinal cord tissue was immersion fixed in 4% paraformaldehyde and embedded in paraffin. Sagittal and transverse serial sections (4–20 μm) were cut. Muscles were removed and snap frozen in liquid nitrogen cooled isopentane. Coronal sections (10 μm) were cut. Myofibers with or without internalized nuclei and fiber cross-sectional areas were quantified^34, 35^ in tissue derived from five mice per group, using ImageJ software v1.43 (NIH, Bethesda, MD, USA). Sciatic nerves were dissected and fixed for at least 2 h in 2.5% of glutaraldehyde, followed by washing of the tissues in 10 mM PBS overnight. The tissues were reduced in 1% osmium tetroxide and following dehydration, embedded in Durcupan. Semithin sections were cut. Hematoxylin–eosin, Holmes–Luxol, Thioflavin S,^36^ as well as ATPase (pH 4.2), Masson's trichrom and *para*-phenylenediamine staining were performed according to standard protocols.^37^ Fibrillar tau pathology was assessed by Gallyas silver staining. Antibodies used for immunohistochemistry are listed under Supplementary Experimental Procedures.

### Electron microscopy

Mice were anesthetized with a mixture of ketamine (100 mg kg^−1^) and xylazine (10 mg kg^−1^) intraperitoneally, injected with sodium pentobarbital (100 mg kg^−1^) and transcardially perfused with PBS, followed by perfusion with 2% paraformaldehyde and 2% glutaraldehyde. Brains and spinal cords were removed and postfixed for 1 h, followed by rinsing of the tissues in 10 mM PBS. The tissues were reduced in 1% osmium tetroxide and 1.5% potassium ferrocyanide and, following dehydration, embedded in Epon. Ultrathin sections from selected areas were cut with a microtome (Ultracut E; Leica Microsystems GmbH, Wetzlar, Germany), collected on single-slot grids and stained in 6% uranyl acetate. Sections were examined and photographed with a Morgagni FEI 80kV electron microscope (FEI Company, Eindhoven, The Netherlands).

### Sarkosyl extraction and western blotting

Following PBS perfusion, one half of the mouse brain was dissected into forebrain and brainstem and frozen in liquid nitrogen. Brain tissue was homogenized 1:10 (w/v) in Tris-buffered saline Complete buffer (20 mM Tris, pH 7.5, 137 mM NaCl, 1 tablet of complete mini protease inhibitor cocktail tablets) and the samples were aliquoted. Sarkosyl extraction was performed as previously described.^23^ Briefly, the brain tissue was homogenized in A68 buffer (0.5 ml of 800 mM NaCl, 10% sucrose, 10 mM Tris-HCl, pH 7.4, 1 mM EGTA) using a Kinetica polytron. Samples were centrifuged at 5000 *g* for 15 min. The supernatant was collected and sarkosyl added to 1%, followed by shaking for 1 h. The samples were then centrifuged at 80 000 *g* for 30 min and the pellet resuspended in 150 μl g^−1^ of 50 mM Tris-HCl, pH 7.4. Western blots were performed under nonreducing conditions by using samples composed of an appropriate amount of protein, 5 μl NuPAGE LDS sample buffer and deionized water. Additional application of 2 μl NuPAGE reducing agent was used to obtain reducing conditions. Antibodies used for western blotting are listed under Supplementary Experimental Procedures.

### Behavioral assessment

Motor behavior, including gait ataxia, tremor and hindlimb reflexes, was assessed. Quantitative motor testing was performed by the grid test in which mice were placed on a vertical mesh grid and the latency to fall off from the grid was recorded for 3 min.

For object recognition test, mice were placed in a squared open field box (48 × 48 × 40 cm) under dim light conditions. Mice were let freely to explore the box during 3 consecutive days for 15 min, until no signs of stress were present (habituation phase). During the following 2 days, two identical objects were introduced at diagonal corners of the field for training sessions of 10 min duration (training phase). Training was halted when the mice had closely explored the objects for 20 s.^38^ Next day, the animals' short-term memory was tested (test phase) by replacing one of the familiar objects with a novel one, and the time spent exploring each object during a period of 6 min was video recorded. Video scoring was done by a researcher blind to the genotype, and as exploration criteria nose sniffing/touching of the object at 2 cm or less distance^38^ were used. For both training and test phases, 10 cm high objects composed of the same material were used, and the position of the novel and familiar objects were randomized across groups.

### Statistics

Statistical analysis was performed using one-way analysis of variance followed by Bonferroni's multiple comparison test and Student's *t*-tests with Graphpad Prism software Version 5.0a (GraphPad Software, La Jolla, CA, USA). *P*-values are reported and outlined as follows: *P<0.05, **P<0.01 and ***P<0.001. The mean and s.d. are indicated.

## Results

### Inducible expression of Δtau results in mild motor palsy, memory dysfunction and pretangle pathology

**Figure 1:**
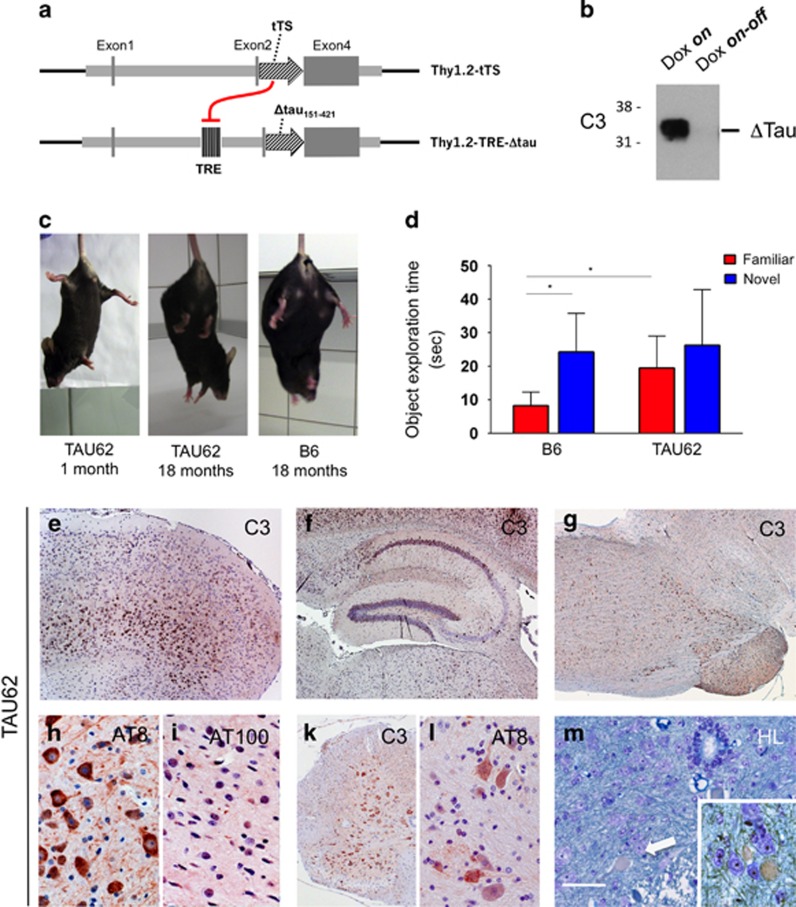
TAU62 mice express Δtau, develop a mild motor phenotype, memory deficits and pretangle pathology. (**a**) Expression constructs. In the presence of doxycycline, 3R tau_151–421_ (Δtau) is expressed. In the absence of doxycycline, tTS (tetracycline-controlled transcriptional silencer) binds to TRE (tetracycline-responsive element), preventing the expression of Δtau. (**b**) Western blot of brain using anti-tau antibody C3 of a 1-month-old TAU62 mouse under doxycycline (Dox *on*) and 3 days after doxycycline withdrawal (Dox *on-off*). (**c**) Tail suspension test on young TAU62 mouse (1 month), aged B6 mouse (18 months) and aged TAU62 mouse (18 months). (**d**) Object recognition test. Object exploration time of adult TAU62 mice (aged 6 months) and their C57Bl6 littermates. (**e–m**) Histology of TAU62 mice aged 12 months (**e–g**) and 18 months (**h–m**). Immunohistochemistry with C3 of somatomotor cortex and orbital area (**e**), hippocampus (**f**), brainstem with tegmental reticular nucleus (**g**) and spinal cord (**k**). Immunohistochemistry of brainstem with AT8 (**h**) and AT100 (**i**); immunohistochemistry of spinal cord with AT8 (**l**). Holmes–Luxol (HL) staining shows the presence of spheroids in spinal cord (arrow; inset) (**m**). The scale bar in (**m**) corresponds to 60 μm in (**h**, **l** and **m**), 80 μm in (**i**), 200 μm in (**e**) and 400 μm in (**f**, **g** and **k**). *P<0.05.

To study the interplay of truncated and full-length human tau *in vivo*, we first generated an inducible transgenic mouse model (line TAU62) overexpressing wild-type 3R tau_151–421_ (Δtau). A tetracycline-controlled, neuron-specific Thy1.2 promoter element (Figure 1a) was used to drive expression that ceased completely upon the removal of doxycycline (Figure 1b).

The Δtau expression resulted in a mild motor phenotype starting at 3–6 months of age. At higher ages, tremor and gait ataxia were followed by mild hindlimb paralysis, and TAU62 mice showed an abnormal limb flexion reflex (Figure 1c and Supplementary Figure 1). Indicative of short-term memory deficits, adult TAU62 mice explored a familiar object significantly longer (*P*=0.04) than their C57Bl6 littermates. The latter accurately discriminated familiar from novel objects (*P*=0.02, Figure 1d).

The Δtau was expressed throughout the central nervous system, including cerebral cortex (Figure 1e), hippocampus (Figure 1f) and brainstem (Figure 1g), comparable to the expression patterns in other transgenic lines using the Thy1.2 promoter.^32^

Pretangle pathology, defined as tau hyperphosphorylation at the AT8 epitope, persisted in aged TAU62 mice (Figure 1h). In contrast to rat models overexpressing truncated tau,^39^ TAU62 mice did not develop tau tangles and showed no hyperphosphorylation of late epitopes, such as AT100, consistent with the absence of tau filaments (Figure 1i). In addition, Δtau was robustly expressed in spinal cord neurons (Figure 1k) where AT8-positive tau accumulated in motor neurons (Figure 1l). Occasional axonal spheroids were seen by Holmes–Luxol staining (Figure 1m). These findings are comparable to those obtained in aged ALZ17 mice.^31^

### Co-expression of Δtau and full-length four-repeat human mutant P301S tau causes early, but reversible, nerve cell dysfunction

**Figure 2:**
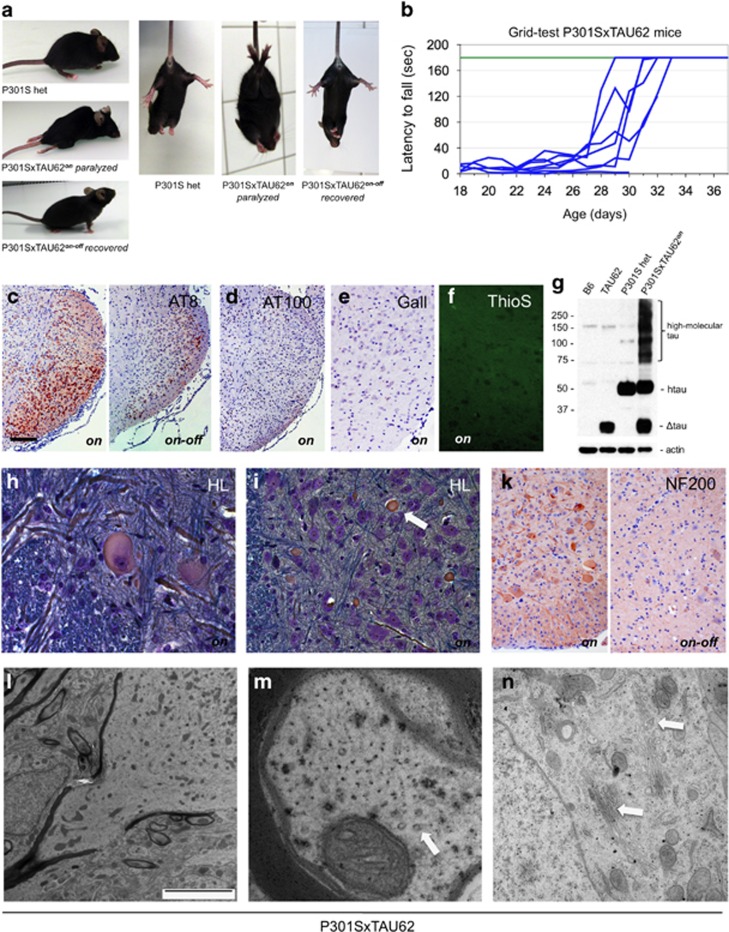
Co-expression of 4R P301S tau and Δtau (P301SxTAU62 mice) causes nerve cell dysfunction that is reversible upon cessation of Δtau expression. Paralysis is associated with the presence of soluble high-molecular-weight tau in the absence of sarkosyl-insoluble tau and tau filaments. Paralyzed mice exhibit axonal accumulations of neurofilaments and mitochondria. (**a**) Heterozygous P301S mouse (aged 3 weeks); paralyzed (aged 3 weeks) and recovered (3 weeks after cessation of Δtau expression) P301SxTAU62 mice (see also Supplementary Videos S1 and S3). (**b**) Recovery of motor function was assessed by a grid test of P301SxTAU62 mice following the removal of doxycycline at 21 days of age (blue lines). Motor function of heterozygous P301S tau littermates (green line, *n*=8). (**c**) Immunohistochemistry with AT8 of the tegmental reticular nucleus of the brainstem of paralyzed (‘*on*') and recovered (‘*on-off*') P301SxTAU62 mice; immunohistochemistry with AT100 (**d**), Gallyas–Braak silver (**e**) and Thioflavin S staining (**f**) of the reticular nucleus of paralyzed mice. (**g**) Western blot with human-specific anti-tau antibody HT7 of brainstem tissue from nontransgenic (B6), TAU62, P301S and P301SxTAU62 mice. (**h** and **i**) Holmes–Luxol (HL) staining of spinal cord of paralyzed 3-week-old P301SxTAU62 mice. The arrow in (**i**) points to a spheroid; (**k**) immunohistochemistry of paralyzed (‘*on*') and recovered (‘*on-off*') mice using antibodies against the 200 kDa subunit of neurofilaments (NF200). The scale bar in (**c**) corresponds to 26 μm in (**h**), 40 μm in (**f** and **i**), 80 μm in (**k**), 100 μm in (**e**) and 200 μm in (**c** and **d**). (**l–n**) Electron microscopy of the spinal cord of paralyzed mice. Only a few isolated microtubules are present in axons (**m**, arrow). Fragmented Golgi material is seen in nerve cell bodies (**n**, arrows). The scale bar in (**l**) corresponds to 5 nm in (**l**), 220 nm in (**m**) and 1.4 nm in (**n**).

We crossed TAU62 mice with tau inclusion-developing four-repeat P301S tau mice (383 amino acid tau isoform with P301S mutation).^32^ Surprisingly, P301SxTAU62^*on*^ mice showed a drastic motor phenotype at 3 weeks of age (Supplementary Video S1). In contrast, homozygous P301S tau mice developed immobilizing limb paralysis at ∼5 to 7 months (Supplementary Video S2), whereas heterozygous mice remained ambulatory until up to 16 months of age. In P301SxTAU62^*on*^ mice, motor impairment started with gait ataxia at 9 days and had evolved to a severe palsy by 3 weeks of age. Paralysis was reversible when Δtau expression was halted at 3 weeks of age. P301SxTAU62^*on-off*^ mice recovered from severe palsy, and their gait normalized within 2–3 weeks (Figures 2a and b, Supplementary Video S3).

### Paralysis of P301SxTAU62 mice is associated with the presence of high-molecular-weight tau

In P301S tau mice, paralysis evolves in parallel to tau tangle formation.^32^ It was therefore surprising that paralyzed P301SxTAU62^*on*^ mice showed only mild pretangle pathology, in the absence of tau filaments (Figures 2c–f, for positive controls see Supplementary Figure 2a). However, the presence of soluble high-molecular-weight tau paralleled the motor impairment (Figure 2g and Supplementary Figure 2b). These tau species comprised Δtau as detected by antibody RD3 (Supplementary Figure 2c). There were no tau bands in the sarkosyl-insoluble fraction (Supplementary Figure 2d). Although high-molecular-weight tau forms were absent in young heterozygous P301S tau mice, similar species were observed in aged homozygous mice (Supplementary Figure 2b). After the expression of Δtau had ceased and P301SxTAU62^*on-off*^ mice were moving normally, high-molecular-weight tau was no longer detectable (Supplementary Figure 2b).

### Reversible axonal damage

The Δtau was widely expressed in the spinal cord of P301SxTAU62^*on*^ mice, resulting in a reversible pretangle pathology (Supplementary Figures 2e–g). Spinal cord neurons of paralyzed mice showed signs of severe dysfunction with pathological swelling, chromatolysis (Figure 2h) and axonal damage, with extensive accumulation of neurofilaments, partly in the form of axonal spheroids (Figures 2i and k). Neurofilament accumulation normalized upon cessation of Δtau expression (Figure 2k).

By electron microscopy, spheroids comprised massed, poorly oriented neurofilaments intermixed with multiple small, congested mitochondria (Figure 2l), compatible with axonal transport disruption. Spinal cord axons of paralyzed P301SxTAU62^*on*^ mice contained only sparse microtubules, whereas neurofilaments were abundant (Figure 2m). Widespread fragmentation of the Golgi network was also seen (Figure 2n).

### Reversible disruption of the Golgi network, dysregulation of synaptic proteins and mitochondrial mislocalization

When aged 3 weeks, P301SxTAU62^*on*^ mice exhibited a fragmented and swollen Golgi network in CA1 pyramidal cells (Supplementary Figures 3a, b, d and e). After Δtau expression was halted, the Golgi structure normalized (Supplementary Figures 3c and f). Synaptophysin immunoreactivity accumulated within the soma of pyramidal cells (Supplementary Figures 3g–i), indicative of transport dysfunction. VAMP2 was lost from CA1 dendrites when Δtau was co-expressed with full-length mutant tau (Supplementary Figures 3k–m). Mitochondria reversibly accumulated within the soma of pyramidal cells, as well as in axons (Supplementary Figures 3n–p).

### Reversible neuropathy and myopathy

**Figure 3:**
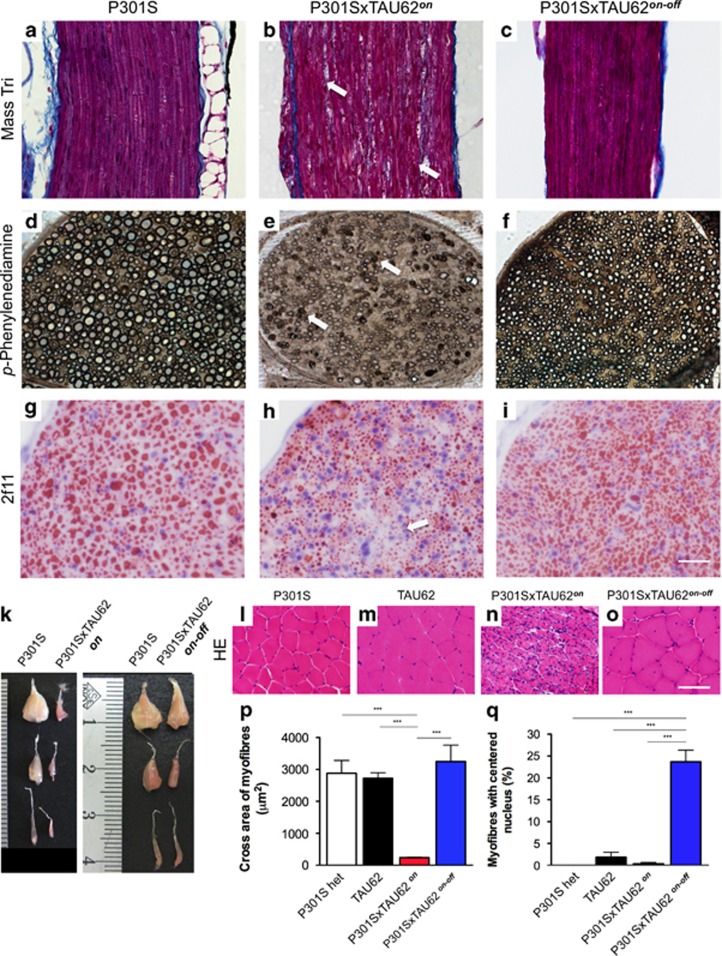
Co-expression of 4R P301S tau and Δtau (P301SxTAU62 mice) causes neuropathy and neurogenic muscle atrophy that are reversible upon cessation of Δtau expression. (**a–i**) Sciatic nerves stained using Masson's trichrom stain (**a–c**), *para*-phenylenediamine (**d–f**) and 2f11 immunohistochemistry (**g–i**). The scale bar in (**i**) corresponds to 50 μm in (**a–c**), 32 μm in (**d-f**) and 25 μm in (**g–i**). (**k**) Macroscopic view of hindlimb muscles. From the top: M. gastrocnemius and M. soleus; M. tibialis anterior; M. extensor digitorum longus. (**l–o**) M. gastrocnemius stained with hematoxylin and eosin (HE). The scale bar in (**o**) corresponds to 100 μm (for **l–o**). Quantification of myofiber area (**p**) and myofibers with internalized nucleus (**q**). P301S: heterozygous mice transgenic for human mutant P301S tau, aged 3 weeks; TAU62: heterozygous mice expressing 3R tau_151–421_, aged 3 weeks; P301SxTAU62^*on*^: paralyzed mice, aged 3 weeks; P301SxTAU62^*on-off*^: recovered mice, 6 weeks after cessation of the expression of Δtau. ***P<0.001.

In paralyzed mice, nerve cell damage was accompanied by an axonal neuropathy (Figures 3a–i and Supplementary Figures 4a–c). The sciatic nerve fibers exhibited vacuolated (Figure 3b) as well as collapsed myelin sheets (Figure 3e), indicating Wallerian degeneration. Neurofilament staining revealed thinned nerve fibers and spotty areas of fiber loss in paralyzed mice (Figure 3h, arrow). Upon motor recovery, myelin debris was no longer detectable and intact nerve fibers of slightly reduced diameter were seen (Figures 3c, f and i). Hindlimb paralysis was associated with muscle wasting (Figure 3k) and marked muscle fiber atrophy (Figure 3n), whereas muscle fibers of heterozygous P301S and TAU62 mice were of normal size (Figures 3k–m and Supplementary Figures 4d and e). Atrophic muscle fibers of paralyzed mice (Figure 3n) were significantly smaller compared with nonparalyzed controls (Figure 3p, *P*<0.001). Both type 1 and 2 fibers were affected and groups of angulated atrophic fibers present, consistent with neurogenic muscle atrophy (Supplementary Figures 4d–g). In parallel with motor improvement, the muscles largely recovered macroscopically (Figure 3k). Recovered mice exhibited partly hypertrophic muscle fibers, with grouping of type 2 fibers, again indicative of neurogenic muscular atrophy (Figure 3o and Supplementary Figure 4g). Upon motor recovery, the percentage of muscle fibers with centralized nuclei was significantly increased in P301SxTAU62^*on-off*^ mice (Figure 3q).

### Co-expression of Δtau and full-length four-repeat human wild-type tau causes early, but reversible, nerve cell dysfunction

**Figure 4:**
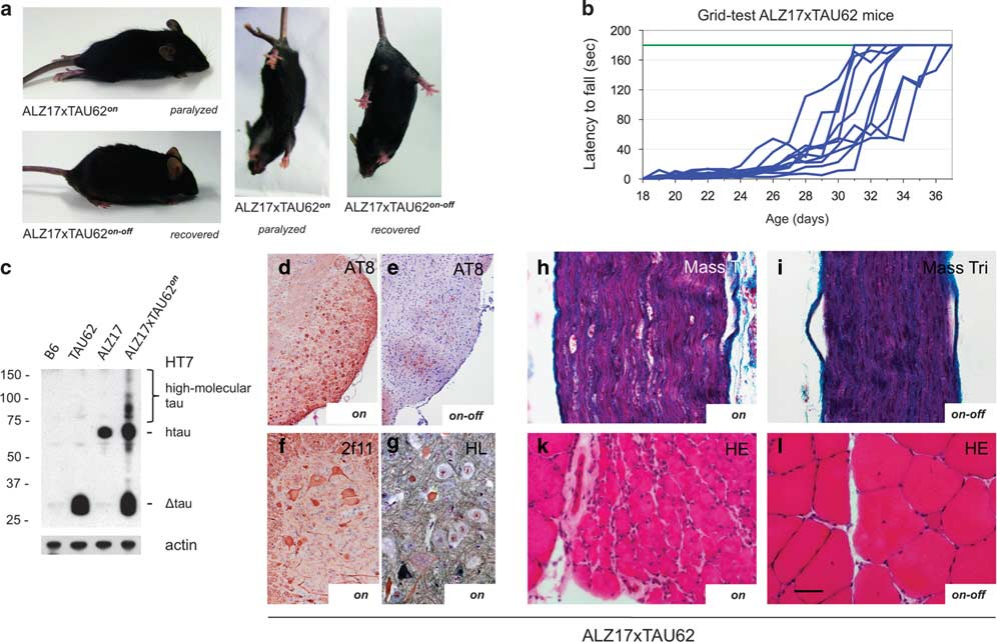
Co-expression of 4R wild-type tau and Δtau (ALZ17xTAU62) causes paralysis and neuropathy that are reversible upon cessation of Δtau expression. (**a**) Paralyzed (aged 3 weeks) and recovered (3 weeks after cessation of Δtau expression) ALZ17xTAU62 mice (see also Supplementary Videos S4 and S5). (**b**) Recovery of motor function as assessed by a grid test of ALZ17xTAU62 mice following the removal of doxycycline between 16 and 20 days of age (blue lines). Motor function of heterozygous ALZ17 littermates (green line) (*n*=6). (**c**) Western blot with HT7 of brainstem tissue from nontransgenic (B6), TAU62, ALZ17 and ALZ17xTAU62 mice. Actin staining was used as the loading control. (**d–l**) Histological analysis of paralyzed (‘*on*', **d**, **f**, **g**, **h** and **k**) and recovered (‘*on-off*', **e**, **i** and **l**) ALZ17xTAU62 mice using anti-tau antibody AT8 (**d** and **e**), anti-neurofilament antibody 2f11 (**f**), Masson's trichrome (**h** and **i**), Holmes–Luxol (HL) (**g**) and hematoxylin–eosin (HE) (**k** and **l**). The scale bar in (**l**) corresponds to 50 μm in (**h**, **i**, **g**, **k** and **l**), 100 μm in (**f**) and 200 μm in (**d** and **e**).

In Alzheimer's disease, tau pathology develops in the absence of *MAPT* mutations. We therefore crossed TAU62 mice with ALZ17 transgenic mice^31^ that express wild-type full-length human four-repeat tau (441 amino acid isoform). ALZ17xTAU62^*on*^ mice showed a similar phenotype to that of P301SxTAU62^*on*^ mice. They developed severe motor palsy within 3 weeks (Figures 4a and b and Supplementary Video S4), and soluble high-molecular-weight tau was present (Figure 4c). Pretangle pathology was accompanied by the accumulation of neurofilaments and the formation of axonal spheroids (Figures 4d–g). Peripheral nerves showed evidence of Wallerian degeneration with ovoid-shaped myelin debris (Figure 4h) with consecutive neurogenic muscle atrophy (Figure 4k). Structural and functional changes were reversible, following the cessation of Δtau expression (Figures 4b, i and l and Supplementary Video S5).

### Co-expression of Δtau and full-length three-repeat human wild-type tau causes early and largely irreversible nerve cell dysfunction

We crossed TAU62 mice with ALZ31 transgenic mice that express wild-type full-length three-repeat human tau (352 amino acid tau isoform). ALZ31xTAU62^*on*^ mice showed severe and early paralysis (Supplementary Figures 5a and b and Supplementary Video S6), developed soluble high-molecular-weight tau (Supplementary Figure 5c) and pre-tangle pathology, as well as neuronal and muscular damage occurred (Supplementary Figures 5d–i). However, unlike what we observed before, most mice failed to recover when Δtau expression was halted.

### Co-expression of two full-length human tau isoforms causes only late nerve cell dysfunction

**Figure 5:**
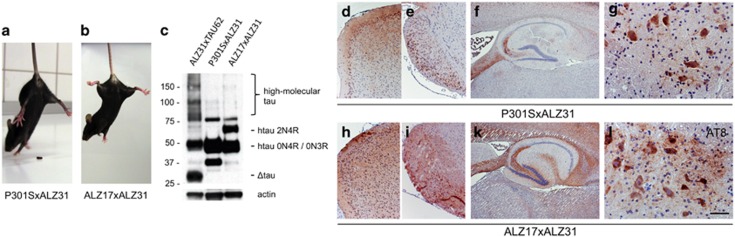
Co-expression of 4R mutant or wild-type tau and 3R wild-type (P301SxALZ31 or ALZ17xALZ31 mice) does not cause paralysis and results in pretangle pathology. (**a** and **b**) Unimpaired P301SxALZ31 and ALZ17xALZ31 mice aged 3 weeks (see also Supplementary Videos S7 and S8). (**c**) Western blot with HT7 of brainstem tissue from paralyzed ALZ31xTAU62 mice aged 3 weeks, unimpaired P301SxALZ31 aged 3 weeks and unimpaired ALZ17xALZ31 mice aged 4 months. Actin staining was used as the loading control. (**d–l**) Immunohistochemistry of 9-month-old P301SxALZ31 mouse and 4-month-old ALZ17xALZ31 mouse with AT8 ((**d** and **h**) cortex, (**e** and **i**) brainstem, (**f** and **k**) hippocampus and (**g** and **l**) spinal cord). The scale bar in (**l**) corresponds to 200 μm in (**d**, **e**, **h** and **i**), 500 μm in (**f** and **k**) and 50 μm in (**g** and **l**).

Slowly progressive, initially mild, motor impairment occurred in mice co-expressing two full-length human tau isoforms (Figures 5a and b). P301SxALZ31 tau mice were still ambulatory at the age of 12 months (Figure 5a and Supplementary Video S7). Similarly, ALZ17xALZ31 tau mice confirmed the absence of severe nerve cell dysfunction (Figure 5b and Supplementary Video S8). Mice from both lines showed robust tau expression, whereas no high molecular tau was detected (Figure 5c and Supplementary Figure 6). They developed only mild motor impairment at the age of 4 months and showed pretangle pathology with extensive AT8 staining (Figures 5d–l).

## Discussion

Here we demonstrate the detrimental interplay between truncated and full-length human tau *in vivo.* Co-expression of truncated tau and full-length wild-type or mutant tau resulted in the formation of soluble, high-molecular-weight tau, severe nerve cell dysfunction, paralysis and marked histopathological changes. Full-length tau and tau truncated at D421 were present in the high-molecular-weight aggregates, consistent with the need for an interaction between the two species. Sarkosyl-insoluble tau or filaments were not present, indicating that nonfilamentous, sarkosyl-soluble, aggregated tau can cause extensive neurotoxicity.

These findings are in agreement with the postulated importance of oligomeric, sarkosyl-soluble tau for the pathogenesis of human tauopathies.^40, 41, 42^ Tau oligomers have been detected in the brains of patients with Alzheimer's disease and progressive supranuclear palsy.^43, 44, 45^ In transgenic mouse models of tauopathies, nerve cell loss and memory deficits can precede detectable filamentous tau pathology.^46, 47, 48^ Moreover, nerve cell loss has been reported in the absence of filaments in tau-overexpressing *Drosophila*,^49^ suggesting that the events that lead from tau accumulation to neurodegeneration may not involve filament formation. Reducing tau overexpression in mice transgenic for human mutant P301L tau has been reported to decrease nerve cell loss, despite the continued formation of tau filaments.^50^

Of the mouse lines transgenic for full-length tau, only that expressing human mutant P301S tau develops sarkosyl-insoluble tau inclusions, neurodegeneration and paralysis. However, these inclusions form only when animals heterozygous for the transgene are more than 12 months old.^51^ When crossed with line TAU62, heterozygous P301S tau mice were paralyzed by the age of 3 weeks, in the absence of sarkosyl-insoluble tau. The same was true of mice transgenic for wild-type 4R tau when crossed with the TAU62 line; wild-type 4R tau-expressing mice do not develop tau inclusions or neurodegeneration.^31^ Whereas soluble oligomeric tau can cause neurotoxicity, it has been reported that either soluble^52^ or insoluble aggregated tau is required for the prion-like propagation of tau assemblies.^53^ It will be interesting to see whether the soluble high-molecular-weight tau described here can seed tau assembly.

Similar to Alzheimer's disease,^54^ axonal spheroids filled with small congested mitochondria and neurofilaments accumulated in the bigenic mouse lines, suggestive of axonal transport deficits. Microtubules were sparse in spinal cord axons, where neurofilaments accumulated, reminiscent of what has been described in Alzheimer's disease^55^ and transgenic mouse models of human tauopathies.^56, 57^ Axonal transport defects have been reported in a broad spectrum of neurodegenerative diseases, including Alzheimer's disease, and the term ‘dysferopathies' has been introduced for this group of disorders.^58, 59^ Paralyzed P301SxTAU62 mice exhibited dislocated and clustered mitochondria, similar to Alzheimer's disease and tauopathy models, where perinuclear mitochondrial clumping correlated with the accumulation of soluble tau species.^60^ Dispersed and swollen Golgi networks, associated with the somatic accumulation of synaptophysin, suggested disrupted cellular transport mechanisms, similar to previous findings in mice transgenic for human mutant P301L tau.^61^ In Alzheimer's disease, Golgi fragmentation has been described in nontangle-bearing neurons,^62^ consistent with the present findings. The neuronal dysfunction present in the absence of tau filaments in our bigenic mouse models may mirror an early stage of tauopathy in Alzheimer's disease.^63^

Although we observed partial nerve fiber loss in paralyzed mice, reflecting toxicity of the oligomeric tau species, the palsy appears mainly attributable to functional neuronal impairment. When Δtau expression was halted, the severe limb paralysis largely improved, despite the continued expression of full-length wild-type 4R or mutant P301S 4R tau. After a lag phase of a few days, paralyzed mice rapidly regained full motor control within 2 days, pointing to a reversible impairment of axonal transport. Rapidly regained nerve fiber function then enables remodeling of atrophic muscle. Only when Δtau had been expressed together with full-length wild-type 3R tau, was paralysis not reversible following the cessation of the expression of truncated tau. Functional recovery thus appears to depend on the presence of mixed 3R/4R tau oligomers. Following cessation of Δtau expression and functional recovery, the high-molecular-weight tau bands disappeared. Co-expression of full-length and Δtau was required, because co-expression of full-length 3R and 4R tau did not cause paralysis.

While the C-terminal end of Δtau constitutes a main tau cleavage site in Alzheimer's disease, its N-terminal end has been set at the structural transition of the proline-rich region to the acidic N-terminal projection domain of tau. This limitation of our model has been unavoidable, as N-terminal tau cleavage sites are yet poorly characterized, and only few N-terminal cleavage sites, located at the beginning of the acidic region of tau, have been confirmed *in situ*.^18, 64, 65^

In conclusion, we show here that an interaction between full-length and Δtau can lead to the formation of neurotoxic tau species that interfere with axonal transport. The reversibility of paralysis upon cessation of truncated tau expression augurs well for the development of new therapies for Alzheimer's disease and other tauopathies.

## References

[bib1] Spillantini MG, Goedert M. Tau pathology and neurodegeneration. Lancet Neurol 2013; 12: 609–622. 23684085 10.1016/S1474-4422(13)70090-5

[bib2] Spires-Jones TL, Hyman BT. The intersection of amyloid beta and tau at synapses in Alzheimer's disease. Neuron 2014; 82: 756–771. 24853936 10.1016/j.neuron.2014.05.004 PMC4135182

[bib3] Frost B, Hemberg M, Lewis J, Feany MB. Tau promotes neurodegeneration through global chromatin relaxation. Nat Neurosci 2014; 17: 357–366. 24464041 10.1038/nn.3639 PMC4012297

[bib4] Rosenmann H. Asparagine endopeptidase cleaves tau and promotes neurodegeneration. Nat Med 2014; 20: 1236–1238. 25375922 10.1038/nm.3749

[bib5] Eisenberg D, Jucker M. The amyloid state of proteins in human diseases. Cell 2012; 148: 1188–1203. 22424229 10.1016/j.cell.2012.02.022 PMC3353745

[bib6] Masters CL, Selkoe DJ. Biochemistry of amyloid beta-protein and amyloid deposits in Alzheimer disease. Cold Spring Harb Perspect Med 2012; 2: a006262. 22675658 10.1101/cshperspect.a006262 PMC3367542

[bib7] Vidal R, Frangione B, Rostagno A, Mead S, Revesz T, Plant G et al. A stop-codon mutation in the BRI gene associated with familial British dementia. Nature 1999; 399: 776–781. 10391242 10.1038/21637

[bib8] Baba M, Nakajo S, Tu PH, Tomita T, Nakaya K, Lee VM et al. Aggregation of alpha-synuclein in Lewy bodies of sporadic Parkinson's disease and dementia with Lewy bodies. Am J Pathol 1998; 152: 879–884. 9546347 PMC1858234

[bib9] Goedert M, Spillantini MG, Del Tredici K, Braak H. 100 years of Lewy pathology. Nat Rev Neurol 2013; 9: 13–24. 23183883 10.1038/nrneurol.2012.242

[bib10] Neumann M, Sampathu DM, Kwong LK, Truax AC, Micsenyi MC, Chou TT et al. Ubiquitinated TDP-43 in frontotemporal lobar degeneration and amyotrophic lateral sclerosis. Science 2006; 314: 130–133. 17023659 10.1126/science.1134108

[bib11] Arai T, Hasegawa M, Akiyama H, Ikeda K, Nonaka T, Mori H et al. TDP-43 is a component of ubiquitin-positive tau-negative inclusions in frontotemporal lobar degeneration and amyotrophic lateral sclerosis. Biochem Biophys Res Commun 2006; 351: 602–611. 17084815 10.1016/j.bbrc.2006.10.093

[bib12] Nonaka T, Kametani F, Arai T, Akiyama H, Hasegawa M. Truncation and pathogenic mutations facilitate the formation of intracellular aggregates of TDP-43. Hum Mol Genet 2009; 18: 3353–3364. 19515851 10.1093/hmg/ddp275

[bib13] Brower CS, Piatkov KI, Varshavsky A. Neurodegeneration-associated protein fragments as short-lived substrates of the N-end rule pathway. Mol Cell 2013; 50: 161–171. 23499006 10.1016/j.molcel.2013.02.009 PMC3640747

[bib14] Gamblin TC, Chen F, Zambrano A, Abraha A, Lagalwar S, Guillozet AL et al. Caspase cleavage of tau: linking amyloid and neurofibrillary tangles in Alzheimer's disease. Proc Natl Acad Sci USA 2003; 100: 10032–10037. 12888622 10.1073/pnas.1630428100 PMC187753

[bib15] Rissman RA, Poon WW, Blurton-Jones M, Oddo S, Torp R, Vitek MP et al. Caspase-cleavage of tau is an early event in Alzheimer disease tangle pathology. J Clin Invest 2004; 114: 121–130. 15232619 10.1172/JCI20640 PMC437967

[bib16] de Calignon A, Fox LM, Pitstick R, Carlson GA, Bacskai BJ, Spires-Jones TL et al. Caspase activation precedes and leads to tangles. Nature 2010; 464: 1201–1204. 20357768 10.1038/nature08890 PMC3091360

[bib17] Khurana V, Elson-Schwab I, Fulga TA, Sharp KA, Loewen CA, Mulkearns E et al. Lysosomal dysfunction promotes cleavage and neurotoxicity of tau *in vivo*. PLoS Genet 2010; 6: e1001026. 20664788 10.1371/journal.pgen.1001026 PMC2904797

[bib18] Horowitz PM, Patterson KR, Guillozet-Bongaarts AL, Reynolds MR, Carroll CA, Weintraub ST et al. Early N-terminal changes and caspase-6 cleavage of tau in Alzheimer's disease. J Neurosci 2004; 24: 7895–7902. 15356202 10.1523/JNEUROSCI.1988-04.2004 PMC6729917

[bib19] Matsumoto SE, Motoi Y, Ishiguro K, Tabira T, Kametani F, Hasegawa M et al. The twenty-four KDa C-terminal tau fragment increases with aging in tauopathy mice: implications of prion-like properties. Hum Mol Genet 2015; 24: 6403–6416. 26374846 10.1093/hmg/ddv351

[bib20] Henriksen K, Wang Y, Sorensen MG, Barascuk N, Suhy J, Pedersen JT et al. An enzyme-generated fragment of tau measured in serum shows an inverse correlation to cognitive function. PLoS One 2013; 8: e64990. 23717682 10.1371/journal.pone.0064990 PMC3661565

[bib21] Avila J. Alzheimer disease: caspases first. Nat Rev Neurol 2010; 6: 587–588. 21048797 10.1038/nrneurol.2010.157

[bib22] Zhang Z, Song M, Liu X, Kang SS, Kwon IS, Duong DM et al. Cleavage of tau by asparagine endopeptidase mediates the neurofibrillary pathology in Alzheimer's disease. Nat Med 2014; 20: 1254–1262. 25326800 10.1038/nm.3700 PMC4224595

[bib23] Delobel P, Lavenir I, Fraser G, Ingram E, Holzer M, Ghetti B et al. Analysis of tau phosphorylation and truncation in a mouse model of human tauopathy. Am J Pathol 2008; 172: 123–131. 18079436 10.2353/ajpath.2008.070627 PMC2189621

[bib24] Lin WL, Dickson DW, Sahara N. Immunoelectron microscopic and biochemical studies of caspase-cleaved tau in a mouse model of tauopathy. J Neuropathol Exp Neurol 2011; 70: 779–787. 21865886 10.1097/NEN.0b013e31822ac9c9 PMC3162471

[bib25] Goedert M, Wischik CM, Crowther RA, Walker JE, Klug A. Cloning and sequencing of the cDNA encoding a core protein of the paired helical filament of Alzheimer disease: identification as the microtubule-associated protein tau. Proc Natl Acad Sci USA 1988; 85: 4051–4055. 3131773 10.1073/pnas.85.11.4051 PMC280359

[bib26] Wischik CM, Novak M, Thogersen HC, Edwards PC, Runswick MJ, Jakes R et al. Isolation of a fragment of tau derived from the core of the paired helical filament of Alzheimer disease. Proc Natl Acad Sci USA 1988; 85: 4506–4510. 3132715 10.1073/pnas.85.12.4506 PMC280459

[bib27] Goedert M, Spillantini MG, Cairns NJ, Crowther RA. Tau proteins of Alzheimer paired helical filaments: abnormal phosphorylation of all six brain isoforms. Neuron 1992; 8: 159–168. 1530909 10.1016/0896-6273(92)90117-v

[bib28] Abraha A, Ghoshal N, Gamblin TC, Cryns V, Berry RW, Kuret J et al. C-terminal inhibition of tau assembly *in vitro* and in Alzheimer's disease. J Cell Sci 2000; 113: 3737–3745. 11034902 10.1242/jcs.113.21.3737

[bib29] Spires-Jones TL, Kopeikina KJ, Koffie RM, de Calignon A, Hyman BT. Are tangles as toxic as they look? J Mol Neurosci 2011; 45: 438–444. 21638071 10.1007/s12031-011-9566-7 PMC3173560

[bib30] Wang YP, Biernat J, Pickhardt M, Mandelkow E, Mandelkow EM. Stepwise proteolysis liberates tau fragments that nucleate the Alzheimer-like aggregation of full-length tau in a neuronal cell model. Proc Natl Acad Sci USA 2007; 104: 10252–10257. 17535890 10.1073/pnas.0703676104 PMC1891218

[bib31] Probst A, Gotz J, Wiederhold KH, Tolnay M, Mistl C, Jaton AL et al. Axonopathy and amyotrophy in mice transgenic for human four-repeat tau protein. Acta Neuropathol 2000; 99: 469–481. 10805089 10.1007/s004010051148

[bib32] Allen B, Ingram E, Takao M, Smith MJ, Jakes R, Virdee K et al. Abundant tau filaments and nonapoptotic neurodegeneration in transgenic mice expressing human P301S tau protein. J Neurosci 2002; 22: 9340–9351. 12417659 10.1523/JNEUROSCI.22-21-09340.2002 PMC6758022

[bib33] Luthi A, Putten H, Botteri FM, Mansuy IM, Meins M, Frey U et al. Endogenous serine protease inhibitor modulates epileptic activity and hippocampal long-term potentiation. J Neurosci 1997; 17: 4688–4699. 9169529 10.1523/JNEUROSCI.17-12-04688.1997 PMC6573330

[bib34] Huang C, Tong J, Bi F, Zhou H, Xia XG. Mutant TDP-43 in motor neurons promotes the onset and progression of ALS in rats. J Clin Invest 2012; 122: 107–118. 22156203 10.1172/JCI59130 PMC3248298

[bib35] Demonbreun AR, Fahrenbach JP, Deveaux K, Earley JU, Pytel P, McNally EM. Impaired muscle growth and response to insulin-like growth factor 1 in dysferlin-mediated muscular dystrophy. Hum Mol Genet 2011; 20: 779–789. 21127009 10.1093/hmg/ddq522 PMC3024047

[bib36] Winkler DT, Biedermann L, Tolnay M, Allegrini PR, Staufenbiel M, Wiessner C et al. Thrombolysis induces cerebral hemorrhage in a mouse model of cerebral amyloid angiopathy. Ann Neurol 2002; 51: 790–793. 12112090 10.1002/ana.10210

[bib37] Romeis B. Mikroskopische Technik. Urban u. Schwarzenberg: München, Wien, Baltimore, 1989.

[bib38] Leger M, Quiedeville A, Bouet V, Haelewyn B, Boulouard M, Schumann-Bard P et al. Object recognition test in mice. Nat Protoc 2013; 8: 2531–2537. 24263092 10.1038/nprot.2013.155

[bib39] Filipcik P, Zilka N, Bugos O, Kucerak J, Koson P, Novak P et al. First transgenic rat model developing progressive cortical neurofibrillary tangles. Neurobiol Aging 2012; 33: 1448–1456. 21196063 10.1016/j.neurobiolaging.2010.10.015

[bib40] Lasagna-Reeves CA, Castillo-Carranza DL, Sengupta U, Sarmiento J, Troncoso J, Jackson GR et al. Identification of oligomers at early stages of tau aggregation in Alzheimer's disease. FASEB J 2012; 26: 1946–1959. 22253473 10.1096/fj.11-199851 PMC4046102

[bib41] Blair LJ, Nordhues BA, Hill SE, Scaglione KM, O'Leary JC 3rd, Fontaine SN et al. Accelerated neurodegeneration through chaperone-mediated oligomerization of tau. J Clin Invest 2013; 123: 4158–4169. 23999428 10.1172/JCI69003 PMC3784538

[bib42] Gerson JE, Kayed R. Formation and propagation of tau oligomeric seeds. Front Neurol 2013; 4: 93. 23882255 10.3389/fneur.2013.00093 PMC3713404

[bib43] Maeda S, Sahara N, Saito Y, Murayama S, Ikai A, Takashima A. Increased levels of granular tau oligomers: an early sign of brain aging and Alzheimer's disease. Neurosci Res 2006; 54: 197–201. 16406150 10.1016/j.neures.2005.11.009

[bib44] Patterson KR, Remmers C, Fu Y, Brooker S, Kanaan NM, Vana L et al. Characterization of prefibrillar Tau oligomers *in vitro* and in Alzheimer disease. J Biol Chem 2011; 286: 23063–23076. 21550980 10.1074/jbc.M111.237974 PMC3123074

[bib45] Gerson JE, Sengupta U, Lasagna-Reeves CA, Guerrero-Munoz MJ, Troncoso J, Kayed R. Characterization of tau oligomeric seeds in progressive supranuclear palsy. Acta Neuropathol Commun 2014; 2: 73. 24927818 10.1186/2051-5960-2-73 PMC4229782

[bib46] Oddo S, Caccamo A, Shepherd JD, Murphy MP, Golde TE, Kayed R et al. Triple-transgenic model of Alzheimer's disease with plaques and tangles: intracellular Abeta and synaptic dysfunction. Neuron 2003; 39: 409–421. 12895417 10.1016/s0896-6273(03)00434-3

[bib47] Spires TL, Orne JD, SantaCruz K, Pitstick R, Carlson GA, Ashe KH et al. Region-specific dissociation of neuronal loss and neurofibrillary pathology in a mouse model of tauopathy. Am J Pathol 2006; 168: 1598–1607. 16651626 10.2353/ajpath.2006.050840 PMC1606598

[bib48] Berger Z, Roder H, Hanna A, Carlson A, Rangachari V, Yue M et al. Accumulation of pathological tau species and memory loss in a conditional model of tauopathy. J Neurosci 2007; 27: 3650–3662. 17409229 10.1523/JNEUROSCI.0587-07.2007 PMC6672413

[bib49] Wittmann CW, Wszolek MF, Shulman JM, Salvaterra PM, Lewis J, Hutton M et al. Tauopathy in Drosophila: neurodegeneration without neurofibrillary tangles. Science 2001; 293: 711–714. 11408621 10.1126/science.1062382

[bib50] Santacruz K, Lewis J, Spires T, Paulson J, Kotilinek L, Ingelsson M et al. Tau suppression in a neurodegenerative mouse model improves memory function. Science 2005; 309: 476–481. 16020737 10.1126/science.1113694 PMC1574647

[bib51] Clavaguera F, Hench J, Lavenir I, Schweighauser G, Frank S, Goedert M et al. Peripheral administration of tau aggregates triggers intracerebral tauopathy in transgenic mice. Acta Neuropathol 2014; 127: 299–301. 24362441 10.1007/s00401-013-1231-5 PMC4253855

[bib52] Lasagna-Reeves CA, Castillo-Carranza DL, Sengupta U, Guerrero-Munoz MJ, Kiritoshi T, Neugebauer V et al. Alzheimer brain-derived tau oligomers propagate pathology from endogenous tau. Sci Rep 2012; 2: 700. 23050084 10.1038/srep00700 PMC3463004

[bib53] Clavaguera F, Bolmont T, Crowther RA, Abramowski D, Frank S, Probst A et al. Transmission and spreading of tauopathy in transgenic mouse brain. Nat Cell Biol 2009; 11: 909–913. 19503072 10.1038/ncb1901 PMC2726961

[bib54] Schmidt ML, Lee VM, Trojanowski JQ. Relative abundance of tau and neurofilament epitopes in hippocampal neurofibrillary tangles. Am J Pathol 1990; 136: 1069–1075. 1693468 PMC1877428

[bib55] Cash AD, Aliev G, Siedlak SL, Nunomura A, Fujioka H, Zhu X et al. Microtubule reduction in Alzheimer's disease and aging is independent of tau filament formation. Am J Pathol 2003; 162: 1623–1627. 12707046 10.1016/s0002-9440(10)64296-4 PMC1851211

[bib56] Yoshiyama Y, Zhang B, Bruce J, Trojanowski JQ, Lee VM. Reduction of detyrosinated microtubules and Golgi fragmentation are linked to tau-induced degeneration in astrocytes. J Neurosci 2003; 23: 10662–10671. 14627651 10.1523/JNEUROSCI.23-33-10662.2003 PMC6740917

[bib57] Zhang B, Carroll J, Trojanowski JQ, Yao Y, Iba M, Potuzak JS et al. The microtubule-stabilizing agent, epothilone D, reduces axonal dysfunction, neurotoxicity, cognitive deficits, and Alzheimer-like pathology in an interventional study with aged tau transgenic mice. J Neurosci 2012; 32: 3601–3611. 22423084 10.1523/JNEUROSCI.4922-11.2012 PMC3321513

[bib58] Morfini GA, Burns M, Binder LI, Kanaan NM, LaPointe N, Bosco DA et al. Axonal transport defects in neurodegenerative diseases. J Neurosci 2009; 29: 12776–12786. 19828789 10.1523/JNEUROSCI.3463-09.2009 PMC2801051

[bib59] Vossel KA, Zhang K, Brodbeck J, Daub AC, Sharma P, Finkbeiner S et al. Tau reduction prevents Abeta-induced defects in axonal transport. Science 2010; 330: 198. 20829454 10.1126/science.1194653 PMC3024010

[bib60] Kopeikina KJ, Carlson GA, Pitstick R, Ludvigson AE, Peters A, Luebke JI et al. Tau accumulation causes mitochondrial distribution deficits in neurons in a mouse model of tauopathy and in human Alzheimer's disease brain. Am J Pathol 2011; 179: 2071–2082. 21854751 10.1016/j.ajpath.2011.07.004 PMC3181340

[bib61] Liazoghli D, Perreault S, Micheva KD, Desjardins M, Leclerc N. Fragmentation of the Golgi apparatus induced by the overexpression of wild-type and mutant human tau forms in neurons. Am J Pathol 2005; 166: 1499–1514. 15855649 10.1016/S0002-9440(10)62366-8 PMC1606403

[bib62] Stieber A, Mourelatos Z, Gonatas NK. In Alzheimer's disease the Golgi apparatus of a population of neurons without neurofibrillary tangles is fragmented and atrophic. Am J Pathol 1996; 148: 415–426. 8579105 PMC1861703

[bib63] Khan UA, Liu L, Provenzano FA, Berman DE, Profaci CP, Sloan R et al. Molecular drivers and cortical spread of lateral entorhinal cortex dysfunction in preclinical Alzheimer's disease. Nat Neurosci 2013; 17: 304–311. 24362760 10.1038/nn.3606 PMC4044925

[bib64] Derisbourg M, Leghay C, Chiappetta G, Fernandez-Gomez FJ, Laurent C, Demeyer D et al. Role of the Tau N-terminal region in microtubule stabilization revealed by new endogenous truncated forms. Sci Rep 2015; 5: 9659. 25974414 10.1038/srep09659 PMC4431475

[bib65] Rohn TT, Rissman RA, Davis MC, Kim YE, Cotman CW, Head E. Caspase-9 activation and caspase cleavage of tau in the Alzheimer's disease brain. Neurobiol Dis 2002; 11: 341–354. 12505426 10.1006/nbdi.2002.0549

