# Supplementary material for: Co-expression of truncated and full-length tau induces severe neurotoxicity
Source: Mol Psychiatry. 2016 Feb 2;21(12):1790–8. doi: 10.1038/mp.2015.228 (PMC5116481; doi:10.1038/mp.2015.228)
Supplement: Supplementary Information [file mp2015228x1.docx]

**Supplemental Information**

***Supplementary Videos:***

**Video S1**

A 3-week-old P301SxTAU62*^on^* double transgenic mouse is shown. Note the paralysis of the hindlimbs, while the mouse is still capable of moving by using the forelimbs.

**Video S2**

Video of a 5-month-old homozygous P301S mouse. Homozygous P301S mice develop overt motor impairments starting at ages of 3-4 months. This mouse is walking slowly, however, there is no severe hindlimb palsy occurring in P301S mice up to ages of 5 months.

**Video S3**

The same P301SxTAU62*^on-off^* mouse seen in Video S1 is shown, but now 38 days after Δtau expression has been stopped by withdrawal of doxycycline. The hindlimb palsy is significantly improved and the mouse is again walking almost normally.

**Video S4**

A 3-week-old ALZ17xTAU62*^on^* double transgenic mouse is shown. Note the paralysis of the hind limbs, while the mouse is still capable of moving by using the forelimbs.

**Video S5**

A recovered ALZ17xTAU62*^on-off^* mouse is shown one month after Δtau expression has been stopped by withdrawal of doxycycline.

**Video S6**

Paralyzed ALZ31xTAU62*^on^* mouse aged three weeks.

**Video S7**

12-month-old P301SxALZ31 mouse. Note the normal grid climbing capability of this double transgenic mouse.

**Video S8**

4-month-old ALZ17SxALZ31 mouse. Note the normal grid climbing capability of this double transgenic mouse.

***Supplementary Table and Figures:***

**Table S1**

**
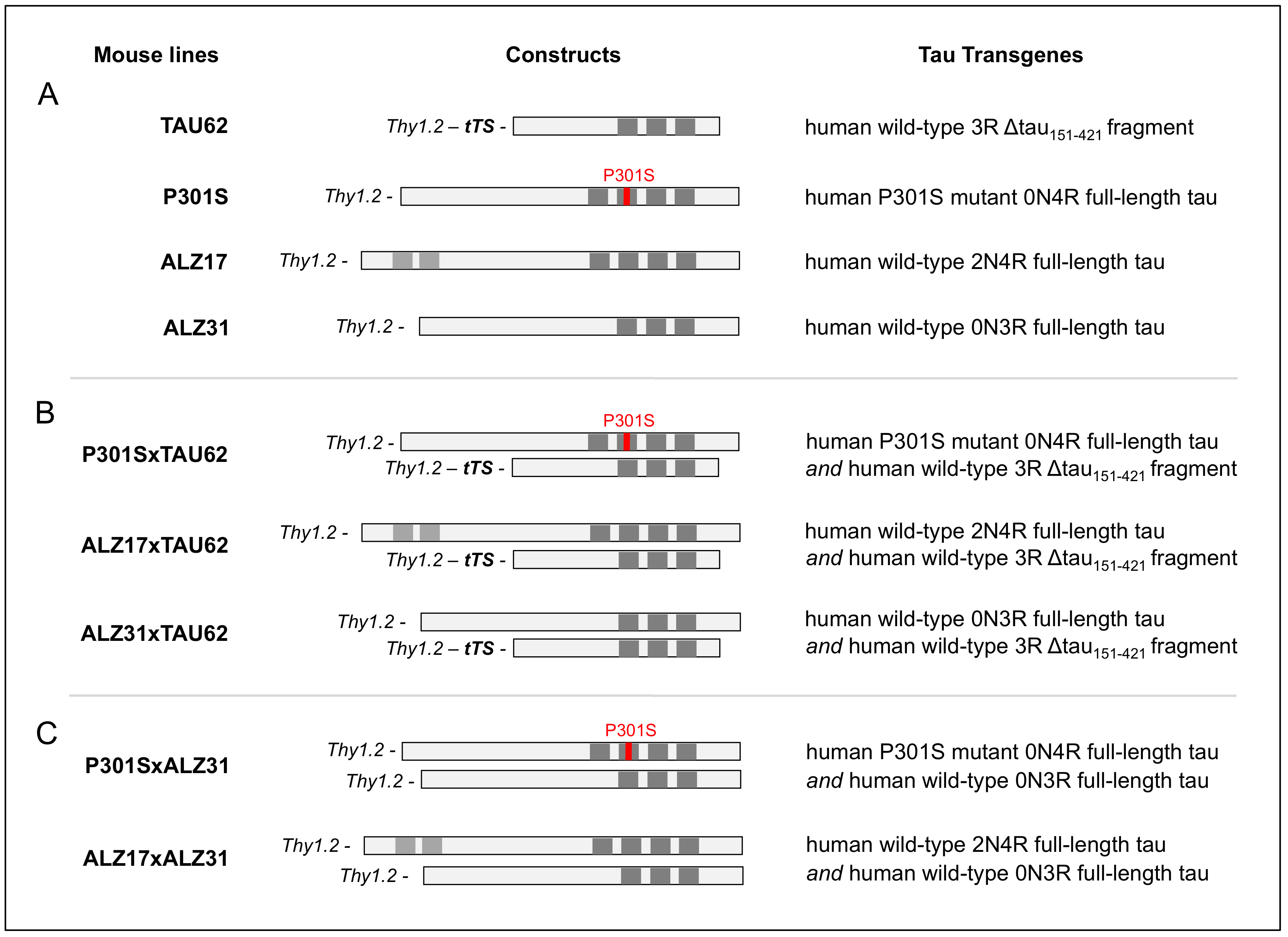
**

(**a-c**) Overview on the transgenic and co-transgenic mouse lines used for the present studies. Tau isoforms expressed are shown. Dark grey boxes indicate the tau repeat domains (3R or 4R isoforms), and light grey boxes N-terminal inserts (e.g. ALZ17 ≙ 2N4R).

The tau cDNA constructs are either driven by a standard Thy1.2 minigene (Thy1.2) or by a modified Thy1.2 minigene that contains a tetracycline controlled transcriptional silencer element (Thy1.2-tTS) in case of the TAU62 mouse. (**a**) shows the tau isoforms expressed in single-transgenic lines. (**b**) shows the tau forms of 3 mouse lines co-expressing full-length tau with Δtau. (**c**) depicts 2 mouse lines co-expressing different full-length tau isoforms.

**Figure S1**

**
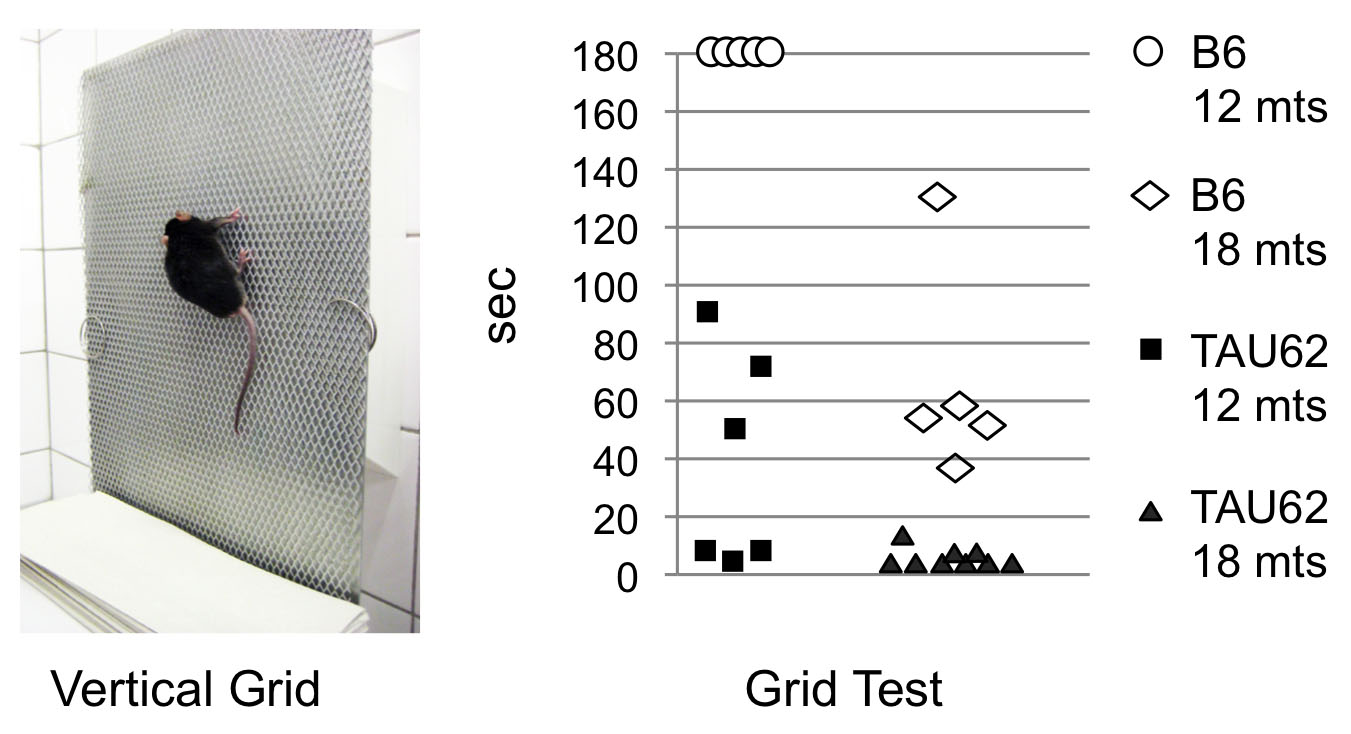
**

*TAU62 mice develop a slowly progressive motor phenotype.*

Motor fitness was assessed on a vertical mesh grid. Time spent on the grid progressively declined in TAU62 mice, while B6 controls were unimpaired at 12 months and showed a mild decline at 18 months of age.

**Figure S2**

**
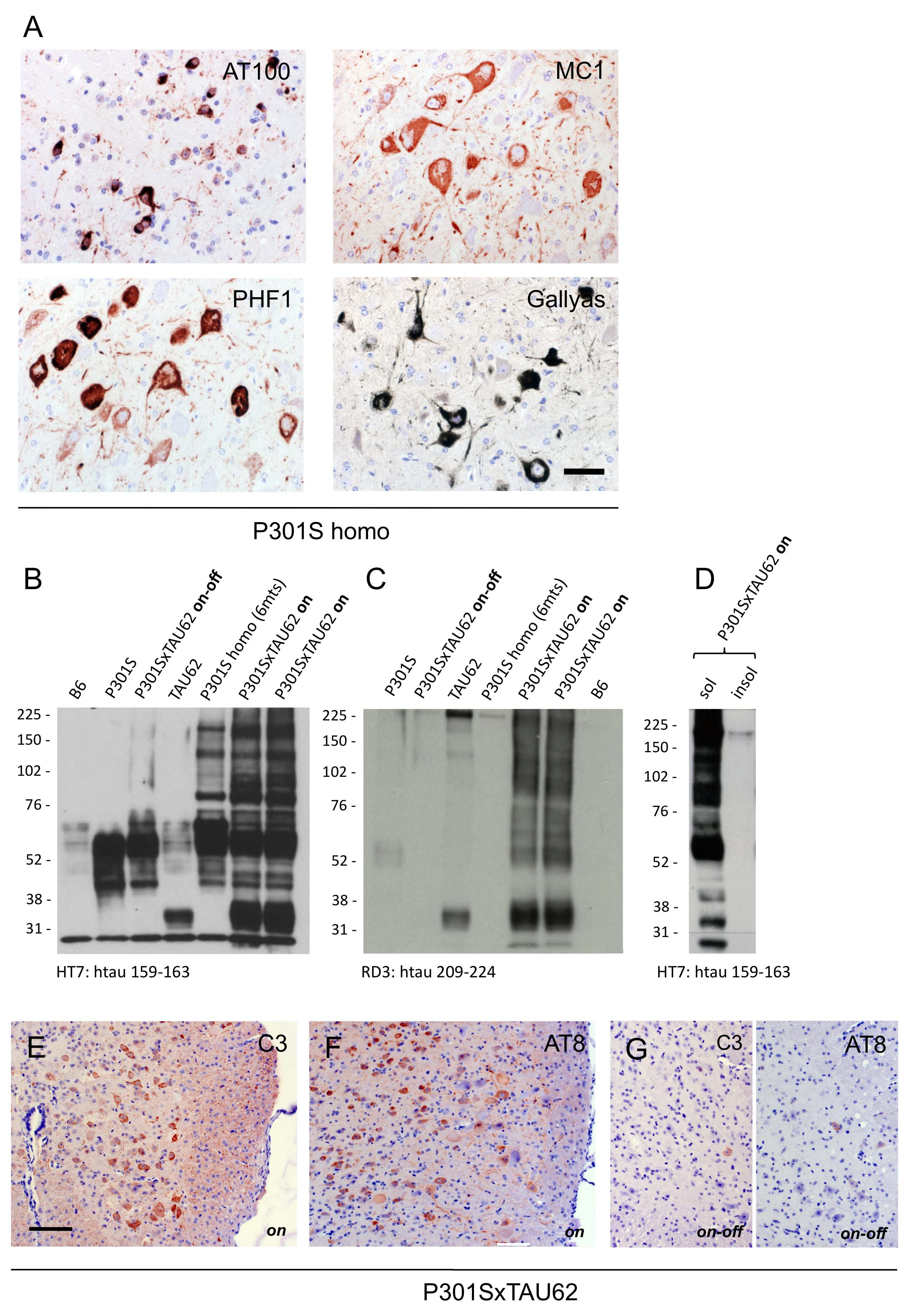
**

(**a**) Extensive hyperphosphorylation of tau was seen in the brainstem of old homozygous P301S mice by staining with antibodies targeting late phospho-epitopes. Multiple tau tangles and granular aggregates were detectable in these mice by Gallyas silver stain. The scale bar in **a** corresponds to 50 μm. (**b**) Western blotting under non-reducing conditions revealed high-molecular tau species in paralyzed P301SxTAU62*^on^* mice (lanes 6&7); similar tau species were seen in aged tangle bearing homozygous P301S mice (lane 5). When tau expression was halted, no more high-molecular tau forms were detectable in P301SxTAU62*^on-off^* mice (lane 3; Western blot performed with anti-tau antibody HT7). (**c**) Staining with the RD3 antibody targeting Asp421 shows the presence of Δtau in the high molecular weight tau species. (**d**) Sarkosyl-extraction detects only soluble tau species in paralyzed P301SxTAU62*^on^* mice (“sol”: sarkosyl-soluble tau; “insol”: sarkosyl-insoluble fraction).

(**e-g**) Δtau was widely expressed in the spinal cord of P301SxTAU62*^on^* mice (**e**) and phosphorylated at the AT8 epitope **(f)**. Upon cessation of Δtau expression, Δtau- and AT8-positive tau was no longer detectable **(g)**. The scale bar in **e** corresponds to 100 μm in **e-g**.

**Figure S3**


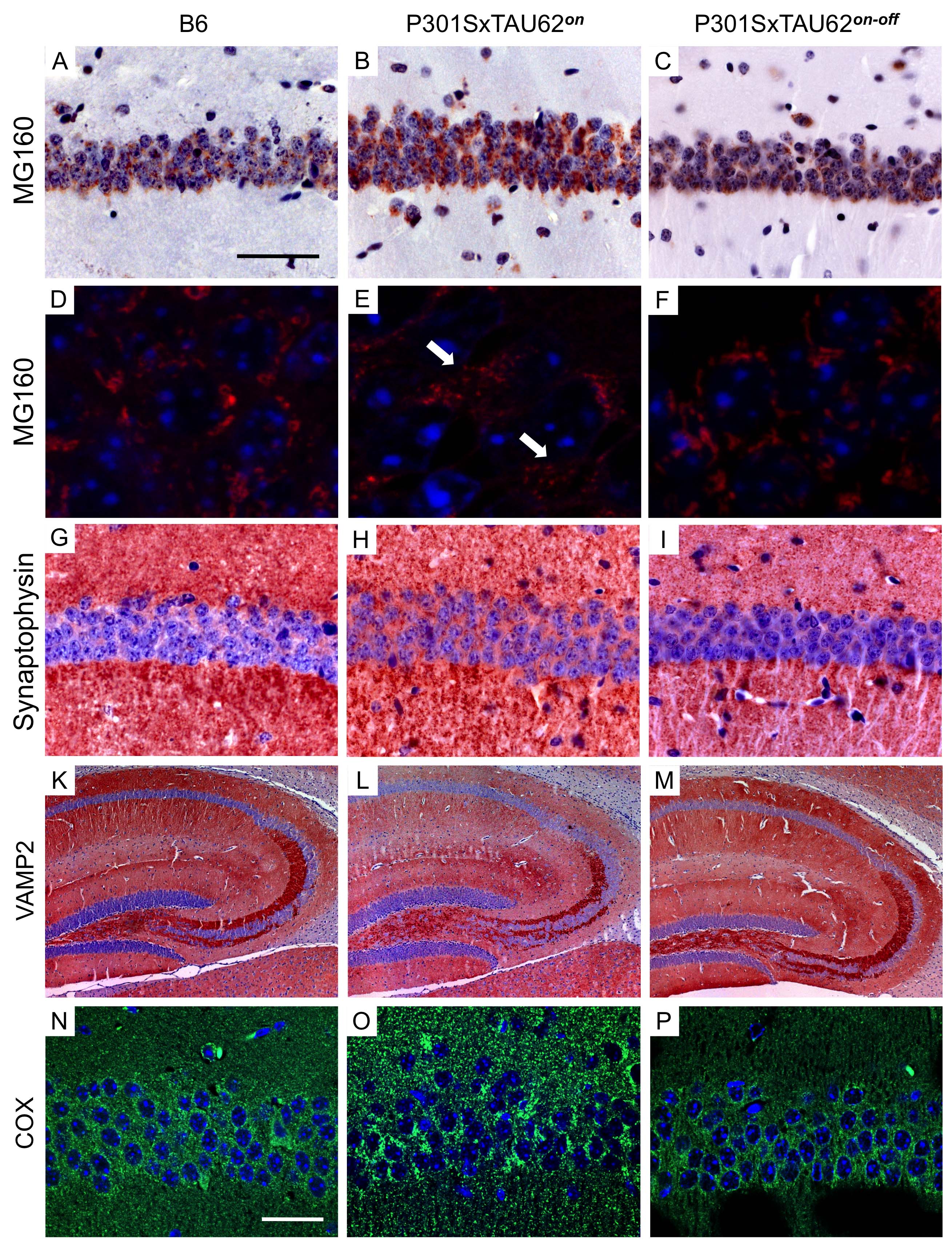


(**a-m**) P301SxTAU62 mice exhibit signs of Golgi disruption, protein missorting and mitochondrial clustering. These signs are reversible upon cessation of Δtau expression. Immunohistochemistry using antibodies against MG160 (**a-f**), synaptophysin (**g-i**), VAMP2 (**k-m**), and cytochrome C oxidase (COX) (**n-p**), in the hippocampus of non-transgenic mice (B6)**,** 3-week-old paralyzed mice (P301SxTAU62^on^) and recovered mice 6 weeks after cessation of Δtau expression (P301SxTAU62^on-off^). The scale bar in **a** corresponds to 19 μm in **d-f**, 63 μm in **a-c** and **g-i,** and 400 μm in **k-m**. The scale bar in **n** corresponds to 30 μm for **n-p**. Arrows in (**e**) indicate fragmented Golgi structures.

**Figure S4**


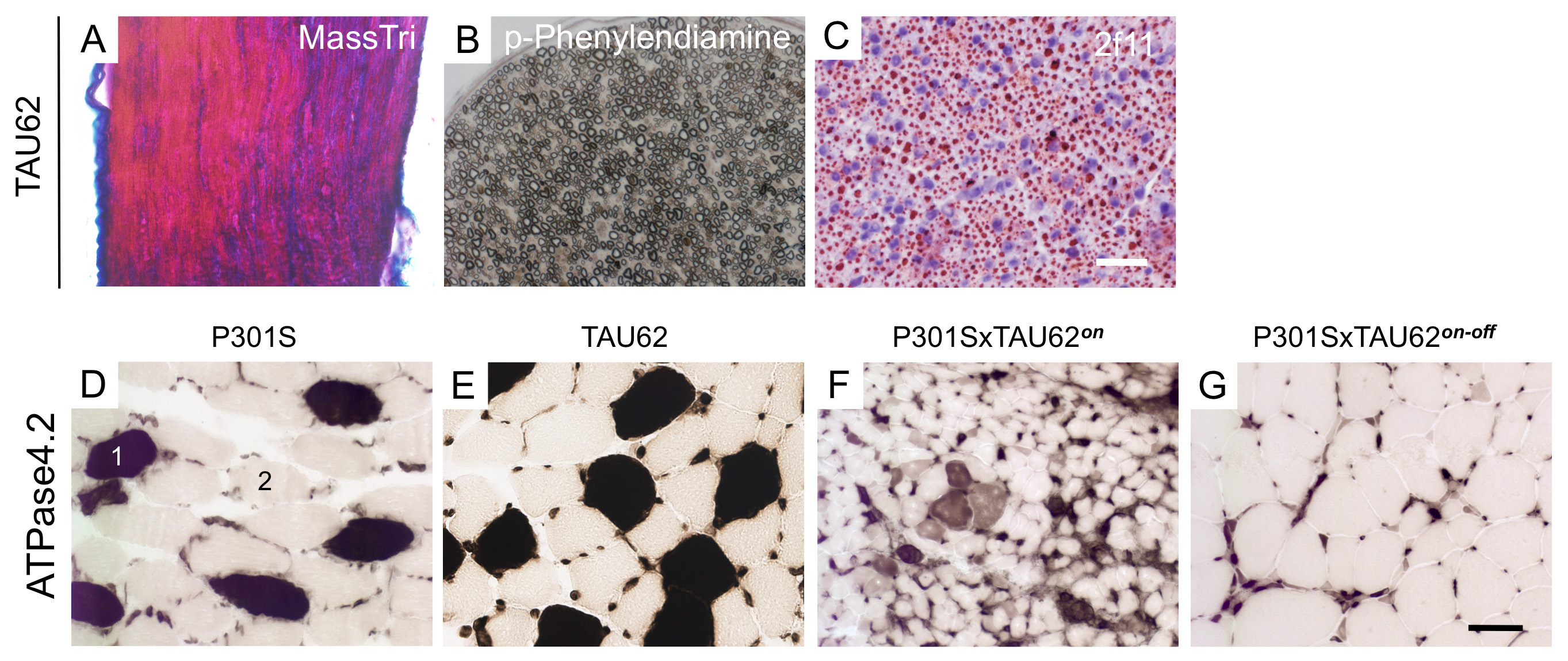


(**a-c**) Young TAU62 mice exhibit normal sciatic nerves (Masson’s trichrom stain (**a**); para-Phenylenediamine (**b**); immunohistochemistry using 2f11 antibody (**c**)). The scale bar in **c** corresponds to 30 μm in **a-c.**

(**d-g**) M. gastrocnemius stained for ATPase (pH 4.2). Dark type 1 fibres (1) and light type 2 fibres (2). The scale bar in **g** corresponds to 50 μm (for **d-g**). P301S: heterozygous mice transgenic for human mutant P301S tau, aged 3 weeks; TAU62: heterozygous mice expressing 3R tau_151-421_, aged 3 weeks; P301SxTAU62*^on^*: paralyzed mice, aged 3 weeks; P301SxTAU62*^on-off^*: recovered mice, 6 weeks after cessation of the expression of Δtau.

**Figure S5**

**
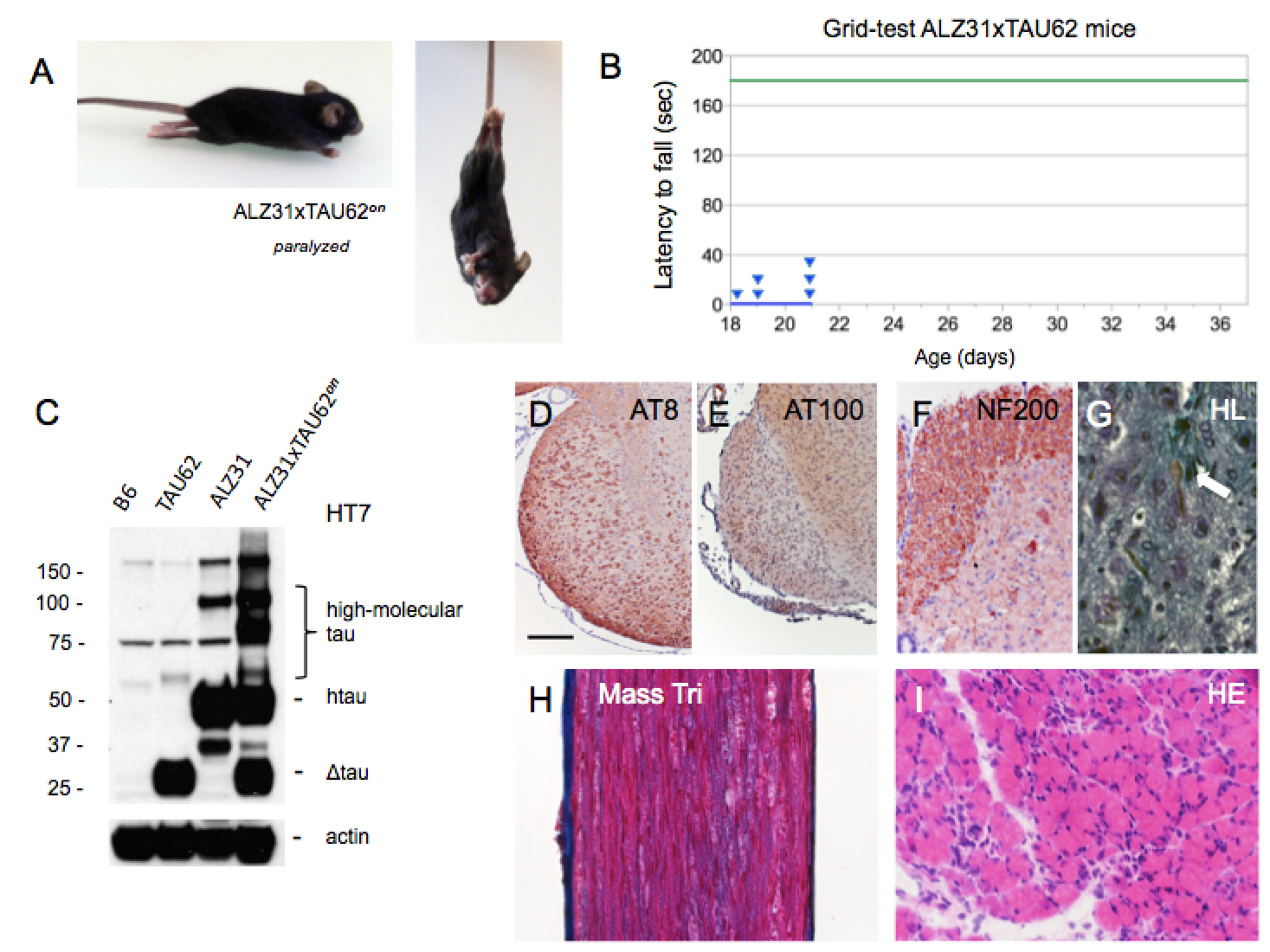
**

**(a-i)** Co-expression of 3R wild-type tau and Δtau (ALZ31xTAU62 mice) causes paralysis and neuropathy, which are not reversed upon cessation of Δtau expression. (**a**) Paralyzed (aged 3 weeks) and non-recovered (3 weeks after cessation of Δtau expression) ALZ31xTAU62 mice (see also video S6). (**b**) Absence of recovery of motor function as assessed by a grid-test of ALZ31xTAU62 mice following the removal of doxycycline between 14 and 16 days (blue line; triangles indicate the times of euthanasia, n=6). Motor function of heterozygous ALZ31 mice (green line, n=7). (**c**) Western blot with HT7 of brainstem tissue from non-transgenic mice (B6), TAU62 mice, ALZ31 mice and ALZ31xTAU62 mice. Actin staining was used as the loading control. (**d-i**) Histological analysis of paralyzed ALZ31xTAU62 mice aged 3 weeks, using AT8 (**d**), AT100 (**e**), NF200 (**f**), Holmes-Luxol (HL) (**g**), Masson’s trichrome (**h**), and Hematoxylin-eosin (HE) stainings (**i**). The arrow in (**g**) points to a spheroid. The scale bar in **d** corresponds to 200 μm in **d** and **e**; 100 μm in **f**; 33 μm in **g**; 50 μm in **h, i**.

**Figure S6**

**
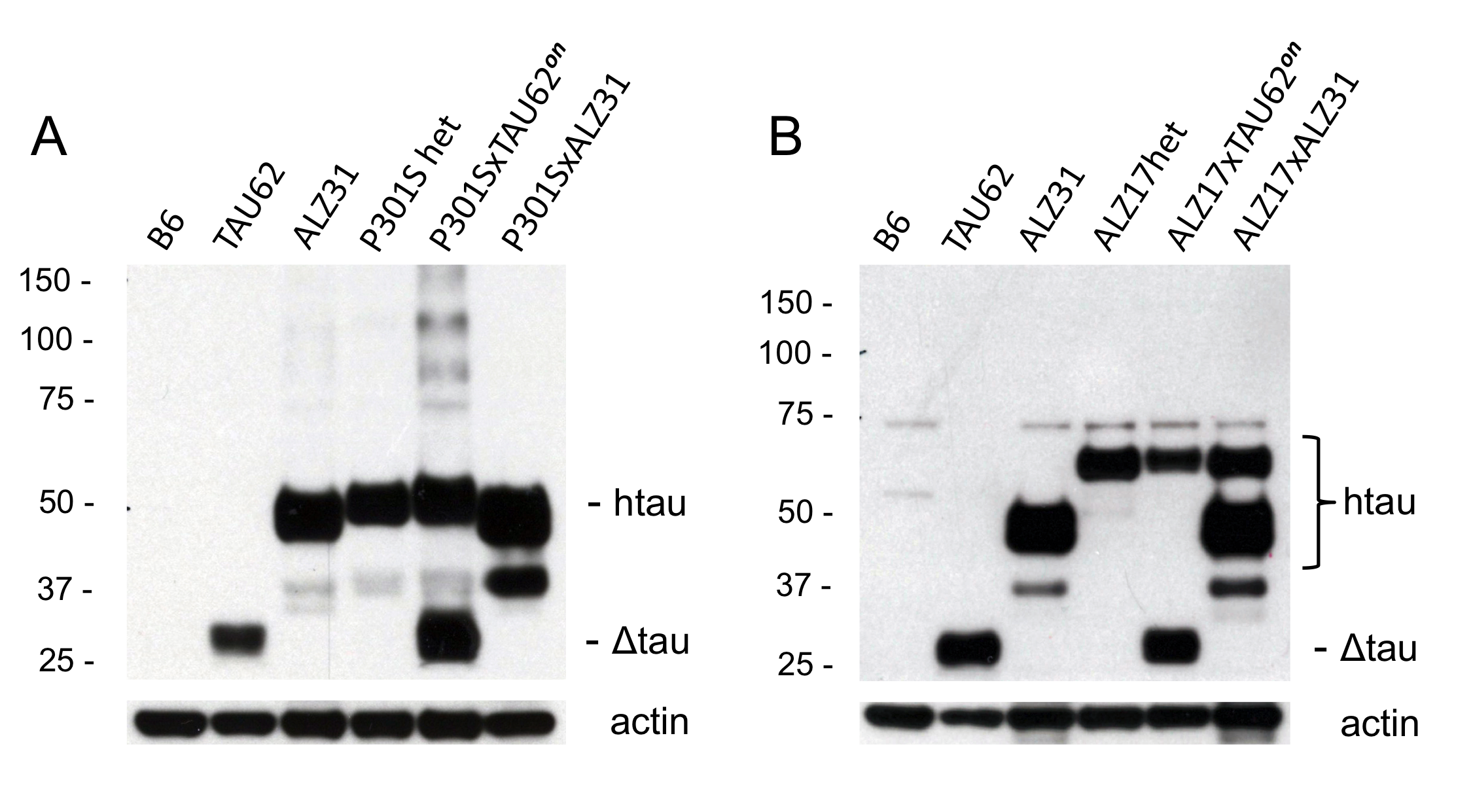
**

(**a,b**) Robust expression of the two full-length tau isoforms in P301SxALZ31 (**a**) and ALZ17xALZ31 (**b**) co-transgenic mice. For comparison, expression of mice co-transgenic for Δtau with full-length tau, as well as the respective single transgenic mice is shown. Western blots run under reducing conditions using HT7 antibody.

**Supplemental Experimental Procedures**

*Antibodies used for immunohistochemistry (IHC) and Western blotting (WB)*

(species is mouse, unless indicated otherwise):

| **Antibody** | **Target** | **Dilution** | **Source** |
| --- | --- | --- | --- |
| HT7 | human tau  aa 159-163 | WB 1:4000  IHC 1:800 | Pierce, Rockford, IL  #MN1000 |
| BR134 | human tau | WB 1:1000 | (1) |
| Tau-C3 | Tau cleaved at residue Asp421 | WB 1:1000  IHC 1:1000 | Santa Cruz Biotechnology, Inc, Dallas, TX  #sc-32240 |
| AT8 | Tau  pSer202/Thr205 | WB 1:1000  IHC 1:800 | Pierce, Rockford, IL #MN1020 |
| AT100 | Tau  pThr212/Ser214 | WB 1:1000  IHC 1:500 | Pierce, Rockford, IL #MN1060 |
| PHF-1 | Tau  pSer396/404 | WB 1:2000  IHC 1:1000 | Peter Davies, Albert Einstein College of Medecine, Bronx, NY |
| MC1 | Tau  aa 5-15, 312-322 | IHC 1:100 | Peter Davies, Albert Einstein College of Medecine, Bronx, NY |
| 2F11 | neurofilament (NF) NF-L, NF-H (70kD) | IHC 1:800 | Dako, Glostrup, DK  #M0762 |
| NF200 | neurofilament (200kD) | IHC 1:100 | (2) |
| GFAP | glial fibrillary acidic protein | IHC 1:500 | Thermo Fisher Scientific Inc., Kalamazoo, MI  #MS-1407-R7 |
| Synaptophysin | synaptophysin | IHC 1:1000 | Millipore Corporation, Billerica, MA #MAB5258 |
| MG160 (rabbit) | Golgi apparatus | IHC 1:1000 | Nicholas Gonatas, Pathology and Laboratory Medicine, University of Pennsylvania, PA |
| VAMP2/Synaptobrevin 2 (rabbit) | transport vesicles | IHC 1:1000 | Synaptic system, Goettingen, Germany  # 104 202 |
| GAPDH (6C5) | GAPDH | WB 1:1000 | Santa Cruz Biotechnology, Santa Cruz, CA, #32233 |
| ß-actin | actin | WB 1:5000 | Sigma-Aldrich, Saint Louis, MO #A5316 |
| Cox subunit 1a | mitochondrial staining | IHC 1:200 | Abcam plc, Cambridge, UK  #ab14705 |

**Supplemental References:**

1. Goedert M, Spillantini MG, Jakes R, Rutherford D, & Crowther RA (1989) Multiple isoforms of human microtubule-associated protein tau: sequences and localization in neurofibrillary tangles of Alzheimer's disease. *Neuron* 3(4):519-526.

2. Probst A*, et al.* (2000) Axonopathy and amyotrophy in mice transgenic for human four-repeat tau protein. *Acta Neuropathol* 99(5):469-481.
